# An open-label, single-arm, phase I/II study of lower-dose decitabine based therapy in patients with advanced hepatocellular carcinoma

**DOI:** 10.18632/oncotarget.3677

**Published:** 2015-03-29

**Authors:** Qian Mei, Meixia Chen, Xuechun Lu, Xiang Li, Feng Duan, Maoqiang Wang, Guangbin Luo, Weidong Han

**Affiliations:** ^1^ Department of Molecular Biology, School of Life Sciences, Chinese PLA General Hospital, Beijing, P. R. China; ^2^ Department of Bio-therapeutic, School of Life Sciences, Chinese PLA General Hospital, Beijing, P. R. China; ^3^ Department of Interventional Radiology, Chinese PLA General Hospital, Beijing, P. R. China; ^4^ Department of Genetics and Genome Sciences, Case Comprehensive Cancer Center, Case Western Reserve University School of Medicine, Cleveland, Ohio, USA

**Keywords:** decitabine, lower dose, advanced hepatocellular carcinoma, hepatotoxicity, phase I/II

## Abstract

**Purpose:**

We conducted this phase I/II clinical trial to determine the safety and efficacy of lower-dose decitabine based therapy in pretreated patients with advanced HCC.

**Experimental Design:**

Patients with advanced HCC were eligible. The administered dose of decitabine was 6 mg/m^2^/d intravenously on days 1 to 5 of a 28-day cycle. Additional therapies were given based on their disease progression status. The endpoint was to ensure the safety, hepatotoxicity, clinical responses, progression-free survival (PFS) and pharmacodynamics assay of lower-dose decitabine.

**Results:**

Fifteen patients were enrolled. The favorable adverse events and liver function profiles were observed. The most beneficial responses were 1 complete response (CR), 6 stable disease (SD) and 8 progressive disease (PD). MRI liver scans post-treatment indicated a unique and specific characteristic. The immunohistochemistry result from the liver biopsy exhibited noteworthy CTL responses. Median PFS was 4 months (95% CI 1.7, 7), comparing favorably with existing therapeutic options. Expression decrement of DNMT1 and global DNA hypomethylation were observed in PBMCs after lower-dose decitabine treatment.

**Conclusion:**

The lower-dose decitabine based treatment resulted in beneficial clinical response and favorable toxicity profiles in patients with advanced HCC. The prospective evaluations of decitabine administration schemes and tumor tissue-based pharmacodynamics effect are warranted in future trials.

## INTRODUCTION

Decitabine (5-Aza-2′-deoxycytidine) is an epigenetic drug that inhibits DNA methylation [1] and has been approved by the Food and Drug Administration for the treatment of myelodysplastic syndrome and acute myelogenous leukemia [2]. The maximally tolerated doses of decitabine for treating solid tumors have proven to be substantially toxic to cancer patients [3, 4]. A clinical trial, conducted in MDS using 0.1-0.2 mg/kg (3.5-7 mg/m^2^) decitabine, revealed the decreased toxicity and complete hematologic and cytogenetic remission, with an overall response rate of 44% [5], suggesting the usage of a very low dosage of decitabine could be a potential compelling and effective form of therapy for solid cancer [3, 6-8]. Based on this hypothesis, we registered a phase I/II clinical trial for lower-dose decitabine therapy, named the “Lower Dose Decitabine Based Therapy in Patients with Refractory and/or Chemotherapy Resistant Solid Tumors or B Cell Lymphomas” (ClinicalTrials.gov identifier NCT01799083).

Hepatocellular carcinoma (HCC) is one of the most common lethal malignancies [9, 10]. The prognosis of HCC is dismal, mainly because diagnosis typically occurs in the advanced stage and there is a lack of feasible treatment modalities [10]. In particular, HCC was selected because of its clinical uniqueness and significance in China. The majority of the Chinese HCCs are associated with viral hepatitis. Importantly, prior trials involving high doses of decitabine did not draw a definite conclusion about the clinical response of HCC and posed a problem to subjects with ultra-hepatotoxicity [11, 12], whereas low-dose decitabine could be a reasonable preferred adoptive choice for the treatment of advanced HCC. Thus, we felt compelled to conduct a thorough verification of the efficacy and safety of lower-dose decitabine therapy in patients with advanced HCC.

The reported lowest total dose of decitabine that has been used to treat a solid tumor was 50 mg/m^2^, but the administration was accompanied by a variety of adverse events (AEs) [7, 11, 12]. Our previous study has confirmed that low-dose decitabine treatment could reverse DNA methylation and induce the re-expression of known epigenetically regulated genes in patients' PBMCs (peripheral blood mononuclear cells) [13]. On that basis, we de-escalated the dose of decitabine in our phase I/II trial to 6 mg/m^2^/d for 5 days of therapy. In this study, we report the results of the efficacy and safety evaluation of the very first phase I/II study of lower-dose decitabine based therapy in Chinese patients with advanced HCC.

## RESULTS

### Patient characteristics

All 15 patients finished 2 to 8 cycles of treatment. The baseline demographics characteristics are shown in Table 1. The median age was 55 years (range, 38-66 years). Performance status ranged from ECOG (Eastern Cooperative Oncology Group) of 0 to 2. Liver function was rated as Child-Pugh Class A in 13 of 15 patients (86.66%). Twelve patients (80%) had chronic HBV infection, and 3 (20%) were negative for HBV and HCV. Eight patients (53.33%) had metastatic disease at the time of study entry. The most frequently affected metastatic sites were the lung (3), adrenal gland (1) and retroperitoneal lymph node (4).

**Table 1:** Baseline characteristics of hepatocellular carcinoma patients

Characteristic	No. of patients	%
**Total No. of patients**	15	100
**Gender**		
Male	14	93.33
Female	1	6.67
**Age, yr**, median (range)	55 (38-66)	
**Finished treatment cycles**		
2	2	13.33
3	2	13.33
>4	11	73.34
**ECOG performance status**		
0	6	40
1	8	53.33
2	1	6.67
**KPS score**		
100	5	33.33
90	10	66.67
**Child-Pugh**		
A	13	86.66
B	2	13.33
**Enrolled tumor stage**		
II	1	6.67
III	6	40
IV	8	53.33
**Max diameter (cm)**		
1-5	6	40
6-10	6	40
>10	3	20
**HCC nodule**		
1-5	12	80
6-10	1	6.67
>10	2	13.33
**HBV infection**	
Yes	12	80
No	3	20
**Tumor burden**		
None	7	46.66
Lung	3	20
Lymph node	4	26.67
Adrenal gland	1	6.67
**Previous local treatment**		
Surgery	7	46.66
TACE	13	86.67
MWA	2	13.33
CIK cell	14	93.33
RFA	11	73.33
Interventional therapy	1	6.67
argon-helium cryosurgery	4	26.67
Tyroserleutide	1	6.67
TAE	13	86.67
Sorafenib	4	26.67
Radiotherapy	5	33.33
PMCT	4	26.67
PRFA	2	13.33
**Liver function**, mean (standard deviation)		
AFP (μg/L)	3748.62 (8399.1)	
ALT (U/L)	64.86 (92.51)	
AST (U/L)	67.82 (111.86)	
Albumin (g/L)	39.45 (4.02)	
Total protein (g/L)	68.15 (5.67)	
γ-GT (U/L)	137.78 (67.24)	

**Figure 1:**
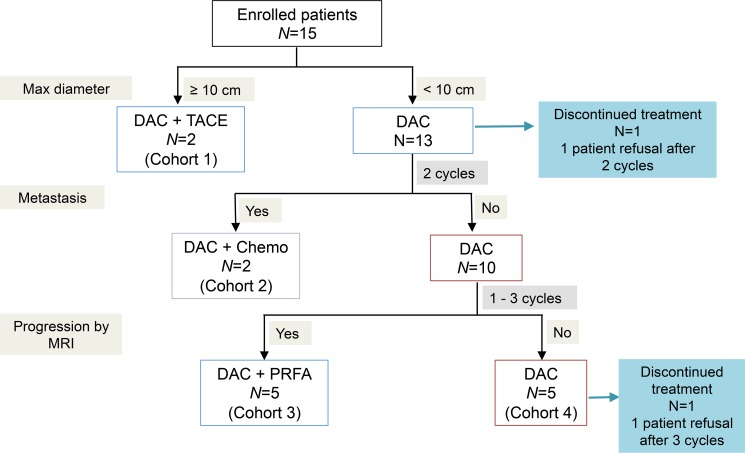
Protocol for lower-dose decitabine based therapy in patients with advanced HCCs DAC, lower-dose decitabine; TACE, transcatheter arterial chemoembolization; PRFA, percutaneous radio-frequency ablation.

**Figure 2:**
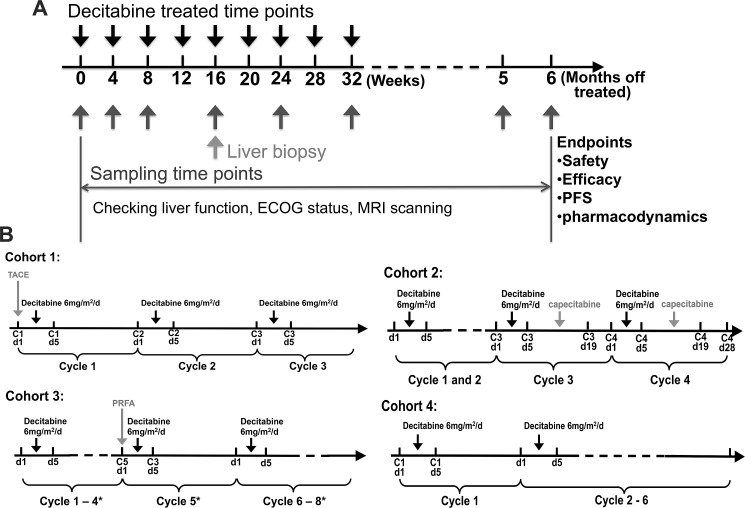
Study design of lower-dose decitabine based therapy **A**. The strategy overview of the present trial of lower-dose decitabine treatment. **B**. Specific dosage regimens and scheme of each cohort. * present the treatment cycles for UPN2 and UPN3, but there should be only two parts of treatment, cycle 1-3 and cycle 5, for UPN6, UPN8, UPN13.

Cohort 1 was opened at the time of study entry based on the overlarge tumor lesion (max diameter ≥ 10 cm) of the two patients UPN11 and UPN 12 (Figures 1 and 2, Table 2). TACE (transcatheter arterial chemoembolization) was regarded as the priority treatment for the HCC patients with larger tumors according to the Clinical Practice Guideline of Primary Liver Cancer of China. Therefore, the 2 patients from cohort 1 underwent TACE before lower-dose decitabine administration to maximize the clinical benefit of the patients from the present trial.

**Table 2:** Summary of hepatocellular carcinoma patients

Patient	Finished cycles	Combined treatment	Finished cycles before other treatment	Response at the end of treatment	Overall response	PFS (M)	Belong to Cohort
UPN1	6	None	—	SD	PD	9	4
UPN2	8	PRFA*	4	PD	Death	4	3
UPN3	8	PRFA	4	PD	PD	4	3
UPN4	4	None	—	SD	SD	17	4
UPN5	4	None	—	PD	PD	2	4
UPN6	4	PRFA	3	PD	PD	1	3
UPN7	4	None	—	CR	CR	11	4
UPN8	4	PRFA	3	SD	PD	5.5	3
UPN9	4	Cape*	2	PD	PD	0	2
UPN10	4	Cape	2	PD	PD	1	2
UPN11	2	TACE*	0	SD	PD	2	1
UPN12	3	TACE	0	SD	PD	5	1
UPN13	4	PRFA	3	SD	SD	4	3
UPN14	3	None	—	PD	Death	0	4
UPN15	2	None	—	PD	PD	0	—

* PRFA, percutaneous radio-frequency ablation; Cape, capecitabine; TACE, transcatheter arterial chemoembolization.

For the two patients from cohort 2, UPN9 and UPN10, strategy was lower-dose decitabine treatment alone until the growth of metastases, then combined with systemic chemotherapy (Figures 1 and 2, Table 2). Based on the clinical guideline of China, the more tolerable first-line chemotherapy regimen, capecitabine, was given orally at the dose of 1.25g/m^2^ 2 times per day. The drug was administrated on 14 consecutive days followed the decitabine treatment in cycle 3 and 4.

Percutaneous radio-frequency ablation (PRFA) was performed on all 5 patients of cohort 3 as required by progression of HCC foci based on MRI (magnetic resonance imaging) scan (Figures 1 and 2, Table 2). Of the 5 enrolled patients of cohort 3, 2 patients, UPN2 and UPN3, received argon-helium cryosurgery followed by 4 cycles of lower-dose decitabine treatment. Meanwhile, microwave ablation (MWA) was performed on the other 3 patients, UPN6, UPN8 and UPN13, after 3 cycles and followed by 1 cycle of decitabation treatment.

The remaining 5 patients was treated for 3 to 6 cycles of lower-dose decitabine monotherapy and assigned to cohort 4 (Figures 1 and 2, Table 2).

### Safety and toxicity

The lower-dose decitabine based treatment was generally well tolerated throughout the study, and none of the 15 patients withdrew from the study because of adverse events. The most commonly reported AEs were hematologic toxicity and gastrointestinal symptoms. Leukopenia was the most common hematologic toxicity, especially within the first 2 cycles (grade 1 to 2; n=10, 62.5%), and white blood cell (WBC) counts improved after adjuvant treatment. Neutropenia was also reported in 3 patients (18.75%, grade 1 to 2). The gastrointestinal symptoms were mainly reported as mild anorexia (n=7, 43.75%), nausea (n=31.25, 30%), vomiting (n=1, 6.25%) and constipation (n=1, 6.25%), and did not required clinical intervention. Some other mild AEs were also observed and are summarized in Table 3. Grade 3 or 4 toxicity was not observed in any of the 15 patients during the entire study. Furthermore, the platelet counts increased with therapy was not statistically significant, while the reduced level of platelet counts of most enrolled patients were noted at baseline (Table 4).

**Table 3:** The incidence of adverse event

Adverse event	Grade 1		Grade 2	
	No.	%	No.	%
**Hematologic**				
Leukopenia	7	46.67	3	20
Neutropenia	2	13.33	1	6.67
**Gastrointestinal**				
Anorexia	5	33.33	2	13.33
Nausea	3	20	2	13.33
Vomiting	1	6.67	0	−
Constipation	1	6.67	0	−
**Other**				
Fatigue	1	6.67	0	−
Hidrosis	2	13.33	0	−
Blurred vision	2	13.33	0	−
Sensory neuropathy	2	13.33	0	−

**Table 4:** Overall Clinical response and liver toxicity profile

Characteristic	Finished treatment cycles									
	Pre		2		3		4		≥ 6	
	No.	%	No.	%	No.	%	No.	%	No.	%
**No. of patient**	15		15		13		11		3	
**Disease status**										
CR	——	—	0	0	1	7.69	1	9.09	0	0
SD			9	60	5	38.46	3	27.27	1	33.33
PD			6	40	7	53.85	7	63.64	2	66.67
**ECOG**										
0	6	40	9	60	8	61.54	7	63.64	1	33.33
1	8	53.33	5	33.33	4	30.77	4	36.36	2	66.67
2	1	6.67	1	6.67	1	7.69	0	0	0	0
**KPS score**										
100	5	33.33	4	26.66	3	23.08	1	9.09	0	0
90	10	66.67	10	66.67	9	69.23	9	81.82	1	33.33
80	0	0	1	6.67	1	7.69	1	9.09	2	66.67
**Child-Pugh**										
A	13	86.67	9	60	7	53.85	7	63.64	2	66.67
B	2	13.33	6	40	6	46.15	4	36.36	1	33.33
**Liver function**, mean (SD)										
AFP (μg/L)	3748.62 (8399.1)		2175.52 (5149.65)		1122.99 (2674.85)		357.8 (507.09)		300.51 (309.82)	
ALT (U/L)	64.86 (92.51)		29.54 (19.1)		30.52 (19.93)		30.61 (14.03)		40.83 (28.65)	
AST (U/L)	67.82 (111.86)		36.71 (19.09)		36.47 (21.58)		35.25 (18.22)		32.07 (16.07)	
Albumin	39.45 (4.02)		37.33 (4.89)		38.89 (5.45)		40.36 (5.45)		37.7 (1.37)	
Total protein	68.15 (5.67)		64.98 (5.33)		67.1 (6.57)		68.13 (5.54)		67.33 (5.15)	
γ-GT	137.78 (67.24)		153.94 (112.09)		168.49 (137.24)		139.46 (110.4)		208 (151.6)	
WBC (10^9^/L)	5.31 (1.46)		4.02 (0.97)		3.87 (1.28)		3.27 (0.84)		3.06 (1.31)	
Plt (10^9^/L)	108.2 (36.78)		132.3 (41.34)		128.9 (46.88)		124.8 (52.48)		152.6 (59.98)	

Because decitabine was the causative event of the compromised liver functions in previous studies [11, 12], we focused on the evaluation of hepatotoxicity in this phase I/II study. The complete liver enzyme profiles were analyzed after every 2 cycles of the therapy (Table 4). The majority patients experienced descending ALT (alanine aminotransferase) and AST (aspartate aminotransferase) levels, but γ-glutamyl transpeptidase (γ-GT) levels were still modestly high in the majority of patients. Intriguingly, the levels of albumin and total protein fluctuated within the normal range in the duration of exposure. The other liver enzymes that leaked into the peripheral blood were not obviously altered by the lower-dose decitabine based treatment that was used in this study. These results suggest that decitabine exerts extremely mild hepatotoxicity effects at the lower dose used in this phase I/II trial and was relatively safe for patients with advanced HCC.

### Efficacy

All 15 eligible patients who received 2 to 8 cycles of treatment patients were assessable for response. In the overall population, one patient achieved a complete response (CR) (6.67%), six patients experienced stable disease (SD) (40%) and 8 had progressive disease (PD) (53.33%) (Table 4), clinical benefit rate (CR + SD rate) was 46.67%. By the end of our data sorting (a follow-up time of ~14 months), the median PFS (progression-free survival) and OS (overall survival) of the corporate patients were 4 and 11 months (95% CI [confidence interval], 1.7–7 months and 8.9–14.6 months) (Supplementary Figure S1).

The PFS of cohort 1 and 3 were basically coincident based their similar therapeutic schemes (lower-dose decitabine combined with palliative local treatment). Patient UPN15 had progressed and exited after 2 cycles treatment. The PFS of 2 patients in cohort 2 was significant shorter than the other cohorts mainly because of the progression of their metastatic lesion (Table 5). Their liver lesions remained SD; if considered liver condition separately, PFS time would be significantly prolonged. Patient UPN2 of cohort 3 received a total of 8 cycles of therapy and achieved stable disease, 6 months after which he died due to clinical deterioration. The extension of PFS of cohort 4 was most significant based on their favourable response to the lower dose decitabine. Patient UPN7 in cohort 4 achieved CR for 11 months. However, the death of UPN14 occurred after 3 cycles of treatment due to disease progression and treatment discontinuation.

**Table 5:** Clinical response and liver toxicity profile of patients of 4 cohorts

Characteristic	Cohort 1		Cohort 2		Cohort 3		Cohort 4	
	Before	After	Before	After	Before	After	Before	After
**No. of patient**	2		2		5		5	
**Disease status^1^**								
CR	—	0	0	0	0	0	1	1
SD	—	2	0	0	0	2	2	2
PD	—	0	2	2	5	3	2	2
**ECOG status**								
0	1	1	1	0	3	2	1	3
1	1	1	1	2	2	3	2	0
2	0	0	0	0	0	0	1	1
**KPS score**								
100	1	1	0	0	1	0	1	1
90	1	1	2	2	3	3	3	2
80	0	0	0	0	1	2	0	1
**Child-Pugh**								
A	2	2	0	0	3	3	4	2
B	0	0	2	2	2	2	0	2
**PFS (M)** mean (SD)	3.5 (2.12)		0.5 (0.71)		3.7 (1.64)		7.8 (6.91)	
**Liver function**, mean (SD)								
AFP (μg/L)	>24200	10442 (12234)	1183.4 (778.67)	1210.6 (632.72)	257.91 (249.74)	272.77 (276.12)	11.68 (13.91)	97.19 (192.22)
ALT (U/L)	207.9 (241.41)	27.05 (2.19)	19.8 (13.29)	31.85 (23.54)	25.34 (11.63)	27.92 (15.91)	59.26 (50.84)	53.56 (22.11)
AST (U/L)	257.49 (296.29)	44.9 (3.96)	42.2 (5.52)	61.55 (30.19)	26.14 (8.65)	27.02 (12.81)	38.72 (25.39)	44.4 (22.65)
Albumin	36.15 (0.21)	36.4 (2.83)	37.5 (5.23)	34.6 (6.51)	39.36 (5.05)	40.32 (3.96)	41.14 (3.51)	40.66 (7.56)
Total protein	64.05 (4.31)	65.25 (2.9)	68 (9.9)	67.35 (9.12)	68.36 (6.84)	69.42 (4.27)	66.42 (6.77)	66.76 (6.97)
γ-GT	200.6 (12.73)	294.7 (152.17)	107.95 (52.26)	133 (60.95)	146.8 (126.87)	185.54 (141.94)	89.08 (68.62)	176.52 (147.64)

**Figure 3:**
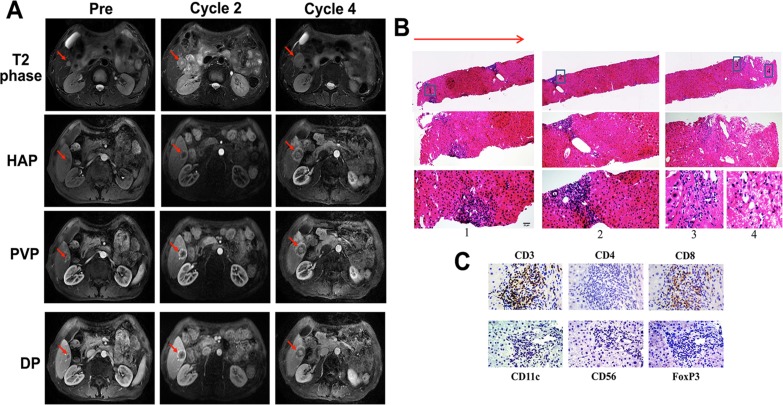
Image of a serial MRI scan and biopsy analysis of the liver tumor for patient UPN1 with disease stabilization **A** The images of T2 phase, HAP (hepatic arterial phase), PVP (portal venous phase) and DP (delayed phase) of MRI after 4 cycles of low-dose decitabine treatment show an enlargement of a liver lesion with a specific characteristic. The red arrows indicate the areas of measurable disease. **B** & **C**. The HE **B**. and immunohistochemical **C**. staining of the biopsy liver tissue of patient UPN1. **B**. The red arrow indicates the direction of the percutaneous CT-guided liver biopsy. The number label indicates the representative area of inflammatory cell infiltration. Magnification, ×10, 20, 40 (from top to bottom). **C**. Magnification, ×40.

**Figure 4:**
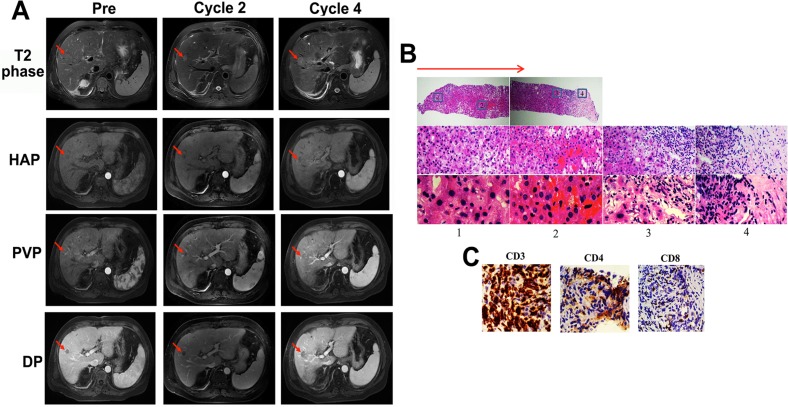
Image of MRI scan and biopsy analysis of the liver tumor for patient UPN2 before palliative local treatment **A**. The images of T2, HVP, PVP and DP phase of MRI after 4 cycles of low-dose decitabine treatment (before palliative local treatment) show an enlargement of a liver lesion with a specific characteristic. The red arrows indicate the areas of measurable disease. **B** & **C**. The HE **B**. and immunohistochemical **C**. staining of the biopsy liver tissue of patient UPN2. B. The red arrow indicates the direction of the percutaneous CT-guided liver biopsy. The number label 1 represents the swollen hepatocytes. Number 2 represents the necrotic area, and numbers 3 and 4 represent the area of inflammatory cell infiltration. Magnification, ×10, 20, 40 (from top to bottom). **C**. Magnification, ×40.

Notably, MRI image of patient UPN1, who received lower-dose decitabine treatment only and achieved SD, displayed a unique characteristic at the T2 phase after the decitabine treatment (Figure 3). The density of the occupying lesions was not homogeneous and appeared as a central opaque area surrounded by a much more translucent texture, similar to the structure of a “fried egg” (Figure 3). The MRI signal indicated that the lesions were organized as tumor, necrosis and inflammation tissue from the inside to the outside. This organization was most obvious in 3 phases (hepatic arterial phase [HAP], portal venous phase [PVP] and delayed phase [DP]) identified by contrast-enhanced MRI imaging (Figure 3). The other patients presented similar MRI scanning images within their SD period, but the organization was not as clear as patient UPN1 (Figure 4).

To confirm the structural characteristics of the occupying lesions that were observed by MRI scanning, percutaneous CT (computerized tomography)-guided liver biopsies should have been performed before other therapeutic regimen added. Unfortunately, only 4 patients (UPN1, UPN2, UPN6 and UPN13) received the biopsy assessment, whereas none of the others consented to a biopsy. The clinicopathologic analysis of liver biopsy of patient UPN1 confirmed the structural characteristics: few to no HCC cells with extensive necrosis surrounded by swollen hepatocyte and exhibited CD8+ cell infiltration, suggesting SD other than PD according to Response Evaluation Criteria in Solid Tumors (RECIST) (Figure 3). The immunohistochemical staining from patients UPN2 presented a similar situation but CD4+ cell infiltration (Figure 4).

### Pharmacodynamics analyses

**Figure 5:**
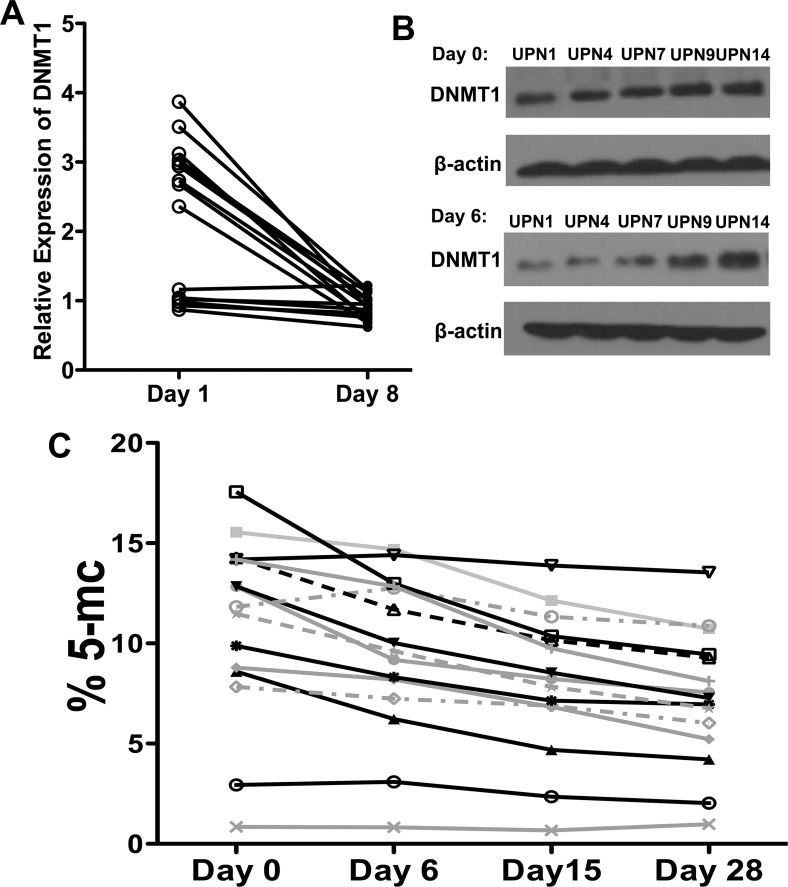
*In vivo* biological activities of lower dose decitabine in the PBMCs **A & B** Western blot analyses of protein expression levels of DNMT1 in PBMCs from all enrolled 15 patients from pre- (day 0) and post-treatment (day 6) of cycle 2. **C**. The % 5-mC of all 15 enrolled patients measured relative to the total cytosine content over time of cycle 2.

To assess the biological activity of the lower-dose decitabine, we investigated the changes in expression levels of the direct target of decitabine *DNMT1* (DNA (cytosine-5-)-methyltransferase 1). Since the pretreatment tumor biopsy material was not available in the current trial, the measurement of DNMT1 expression was performed with peripheral blood mononuclear cells (PBMCs) by western blot analysis. The elevated gene expression of *DNMT1* was observed in PBMCs of 8 patients treated with lower-dose decitabine on day 6 of cycle 2 (Figure 5). Furthermore, the global DNA hypomethylation by decitabine was demonstrated by the downward trend in methylation of LINE-1 repetitive elements (Figure 5). Collectively, our data suggested that the lower dose of decitabine could demethylate and re-express some tumor-related genes, and this at least partially contributes to anti-tumor activity of lower-dose decitabine based therapy in patients with advanced HCC.

## DISCUSSION

In this phase I/II study, we confirmed the safety and efficacy of lower-dose decitabine in heavily pretreated patients with advanced HCC. Decitabine was administered at a dose far below the maximally tolerated dose (6 mg/m^2^/d), which has a confirmed pharmacological demethylation effect on PBMCs [13]. The disease control rate (CR + SD rate) was up to 46.67%. The treatment prolonged PFS and OS to 4 and 11 months in patients with advanced HCC, which is superior to the estimated 3.3 and 9.1 months, respectively [14]. The lower-dose decitabine exhibited a favorable AEs profile for patients with advanced HCC. To the best of our knowledge, this is the first study of the application of lower-dose decitabine, a hypomethylating agent, for antitumor therapy of patients with advanced HCC.

The liver has been noted as the main detoxifying organ for decitabine because of the enrichment of cytidine deaminase (CDA). *Saunthararajah* proposed that in the liver, there might be a reduction of the concentration of decitabine to a sub-therapeutic level [3, 15]. However, these conclusions were based on the existence of the normal tissue architecture of liver, but one of characteristics of HCC is the loss of cell polarity and decrement of liver function [16], and this may result in a deficiency in the CDA enzyme system and prolonged half-life of decitabine in primary HCC patients. The specific SD of liver lesions in cohort 2 indicated that this might be the pivotal factor for clinical effectiveness and favorable hepatotoxicity. The speculation and our results merit additional focused investigation.

A favorable adverse event profile was observed in this phase I/II study, which was highlighted by the finding that only grade 1-2 adverse events were observed. The AEs were predictable and manageable. In most cases of the prior reported studies, the administration of decitabine was either combined with other anticancer agents, or it was administered alone at a relatively high dose [7, 17], which resulted in additional serious grade 3-4 toxicity events. The most common reported AE was leukopenia, consisting with previous studies demonstrating the non-cytotoxic mechanism of decitabine [18]. Whereas, the platelet counts did not increase significantly but fluctuated with the treatment, which might be attributed to the poor liver function and aberrant platelet count at the base line. Furthermore, a lack of direct cytotoxicity from lower-dose decitabine may contribute to a reduction in liver toxicity, and no obvious hepatotoxicity was observed in any of the 15 enrolled patients in our study. Although the cohort of this current study is relative small, the results have clearly demonstrated that the specific treatment regimen is safe with a limited adverse event profile. The single-agent decitabine regimen at the lower dose used in our trial may be an attractive and safe approach.

The proposed mechanism of action of low-dose decitabine is hypomethylation of DNA, which ultimately “normalizes” the gene expression profile of cancer and modulates multiple tumor signaling pathways simultaneously [19, 20]. The epigenetic mechanism of action of lower-dose decitabine is S-phase dependent, requiring overlap between drug exposure time and cancer S-phase entries [21, 22]. Moreover, the rapid disappearance of decitabine has been detected in plasma and demanded a longer duration of therapy due to the short half-life of decitabine [18, 22, 23]. Considering the S-phase dependent mechanism and short *in vivo* half-life, the ideal administration of decitabine should be the long enough duration of therapy to permit all the cancer cells to enter the S-phase of the cell cycle. In this context, the unacceptable hematopoietic toxicity would hamper the clinical application of decitabine [22]. Fortunately, it has been demonstrated that longer infusion schedules of decitabine administration would be as effective as repeated shorter infusions, in light of the asynchronization of cell cycle of cancer cells and the antitumor “memory” response produced by the exposure to low-dose decitabine [20, 24, 25]. In the present trial, a consecutive 5-day intravenous push of lower-dose decitabine was performed during each 28-day treatment cycle, consistent with the traditional administration of S-phase dependent anti-cancer drugs to ensure the safety of the evaluated decitabine therapy. However, as previously described, the subcutaneous administration and/or increased exposure time of decitabine might promise the more favorable clinical benefits and AE profile [18, 23]. The administration of decitabine possessed significant logistic challenges, and the assessment of efficacy of different schemes of lower-dose decitabine are warranted in our now-ongoing phase II study.

Higher proliferation rate was an important hallmark of tumor cells, and the S-phase-specific mechanism of action of lower-dose decitabine has been documented [18, 23]. Based on these, we hypothesized that the DNA incorporation of decitabine might be higher in tumor cells than the surrogate tissues (PBMCs). This should be confirmed by the pharmacodynamics analysis in both tumor tissue samples and PBMCs. The primary design of the current trial was to perform the HCC biopsies after 4 cycles of decitabine treatment, but unfortunately only part of patients consented a biopsy. Moreover, because the pretreatment biopsies were not mandated in this trial, we did not have available pretreatment tumor tissues. Thus, the pharmacodynamics analyses of the tumor tissue samples were not executable, and we will determine the pharmacodynamics effect of lower-dose decitabine in tumor cells of HCC in our future study.

Numerous laboratorial and clinical data have indicated that low-dose decitabine has the ability to affect the activity and differentiation of immune cells and the expression of cancer cell surface makers [24, 26]. Additionally, previous reports demonstrated that 3-5% of the genes modulated by low-dose decitabine in PBMCs were immunomodulatory genes [24]. Thus, we could draw the hypothesis that low-dose decitabine may play a critical role in the epigenetic reprogramming that is related to the immune reconstruction in the tumor bed. The immunohistochemical analysis of the liver biopsy tissue of UPN1 and UPN2 indicated massive inflammatory cell infiltration of tumor bed. The different subtypes of infiltrating T cells (CD8+ and CD4+) may be the foundation of their distinct outcomes (SD and PD). Although the results were not amenable to statistical analysis because of the low patient number, our data were nonetheless supportive of our proposed hypothesis. Our phase I/II study is the first analysis of the *in vivo* effects of decitabine on the immunology of solid tumor patients, but the effect of decitabine needs to be examined and confirmed in future trials with a larger number of patients.

In summary, we confirmed the tolerance and antitumor activity for the lower-dose decitabine based therapy in patients with advanced HCC. This report describes a small phase I/II study, and it is difficult to draw a solid conclusion about the mechanism of action and determine efficacy assessment criteria. This study nevertheless highlights several possible attractive investigational directions of clinical and laboratory decitabine research, and further clinical trials of this lower-dose regimen of decitabine should be considered.

## MATERIALS AND METHODS

### Patients

Written informed consent was obtained from each enrolled patient of this Phase I/II study. Eligible patients were aged from 18 to 85 years old and had pathologically or clinically proven advanced HCC after 1 to 3 prior anticancer regimens were completed. Additional eligibility criteria were as follows: at least one site of radiographically measurable disease of ≥ 1 cm in the largest dimension by a traditional CT scanning technique or ≥ 1 cm in the largest dimension by spiral CT scanning; ECOG performance status of 0 to 2; Child-Pugh class A to B liver disease; and adequate hepatic, hematologic and renal function (WBC ≥ 3000/mm^3^, hemoglobin ≥ 8.0 g/L, platelets ≥ 50000/μL, AST and ALT ≤ 3 times the upper limit of normal [ULN], total bilirubin ≤ 3.0 mg/dL, serum albumin ≥ 2.8 g/dL, serum creatinine ≤ 1.5 times the ULN). The following exclusion criteria were applied: anticancer therapy (including chemotherapy, radiation therapy and immunotherapy) within 3 weeks prior to the first dose; active serious infection; receipt of any other investigational agents or decitabine; severe cardiac insufficiency; history of organ allograft; immunodeficiency; known history of HIV infection; significant neuropathy; pregnancy or lactation; and unsuitability for the trial, based on clinical judgment

### Study design

This phase I/II study protocol conforms to the ethical guidelines of the 1975 Declaration of Helsinki as reflected in a priori approval by the Ethics Committee of the Chinese PLA General Hospital. The study was conducted from August 2012 to June 2014. The present study was undertaken in accordance with the Clinical Practice Guideline of Primary Liver Cancer of China (National Health and Family Planning Commission of the People's Republic of China, 2011). Based on the guideline, in order to ensure the clinical benefits of patients, the patients were divided into four cohorts and given different additional treatments. At least 2 patients were accrued to each cohort (Figure 1). The rationale for the cohorts was as follows: cohort 1, the patients, tumor diameter of whom ≥ 10 cm at the time of study entry, received TACE followed by lower-dose decitabine treatment; cohort 2, systemic chemotherapy with the first-line drugs was given to the patients, who experienced progression of metastasis during the decitabine treatment cycles; cohort 3, PRFA was performed between the cycles of decitabine treatment when the progression was indicated by MRI scan; cohort 4, the lower-dose decitabine monotherapy group. Decitabine (DacoGen, Pharmachemie BV) was administrated at 6mg/m^2^/d by intravenous push on day1 to 5 of each 28-day treatment cycle. Decitabine was stored as a stable freeze-dried powder, reconstituted in 10 mL of sterile water, and diluted to a final volume of 25 mL for injection immediately before use.

### Safety

Treatment of patients will continue until disease progression, unacceptable AEs or patient withdrawal. Safety assessment included physical examination, vital signs, height, weight, ECOG performance status, AEs and laboratory analysis. All evaluations were performed after each treatment cycles (Figure 1). Adverse events were graded according to the National Cancer Institute Common Toxicity Criteria for Adverse Events, version 3.0 (CTCAE).

### Efficacy

MRI was used to confirm the treatment response after every cycle of therapy, and the same imaging modality was used at baseline and follow-ups. In addition, if the physical condition of the patient allowed, a percutaneous CT-guided liver biopsy was performed to further assess responses within the first 30 days after the 4th cycle of treatment. Efficacy variables were evaluated, including the objective response definition, PFS and OS. Tumor responses were assessed in a blinded manner using RECIST 1.0 combined with the pathological analysis of the liver biopsies. PFS was defined as the time from randomization to the first documentation of disease progression or death. OS was defined as the time from the starting treatment to the date of death.

### Pharmacodynamics assay

Whole blood was collected from all enrolled 15 patients prior to treatment and on the day 6, 15 and 28 of cycle 2. The PBMCs were isolated for pharmacodynamics assay. Western blot was used to measure the expression alternation of DNMT1 between the pre- and post-treatment samples. The global methylation was detected using the Global DNA Methylation LINE-1 kit (Active Motif, USA) according to the manufacture's instruction. All data presented reached preset acceptance criteria. Antibody specific to DNMT1 (5032P) was purchased from Cell Signaling Technology (Danvers, MA).

### Statistical analysis

The safety population (all patients who received 2 to 8 cycles of treatment) was used for all analyses. All data were summarized using simple description statistics for the continuous assessment of safety and efficacy for lower-dose decitabine in patients with advanced HCC.

## SUPPLEMENTAl MATERIAL FIGURE AND TABLE


