# Supplementary material for: An open-label, single-arm, phase I/II study of lower-dose decitabine based therapy in patients with advanced hepatocellular carcinoma
Source: Oncotarget. 2015 Mar 29;6(18):16698–711. doi: 10.18632/oncotarget.3677 (PMC4599300; doi:10.18632/oncotarget.3677)
Supplement: Supplementary file 1 [file oncotarget-06-16698-s001.pdf]

An open-label, single-arm, phase I/II study of lower-dose decitabine based therapy in patients with advanced hepatocellular carcinoma

Supplementary Material

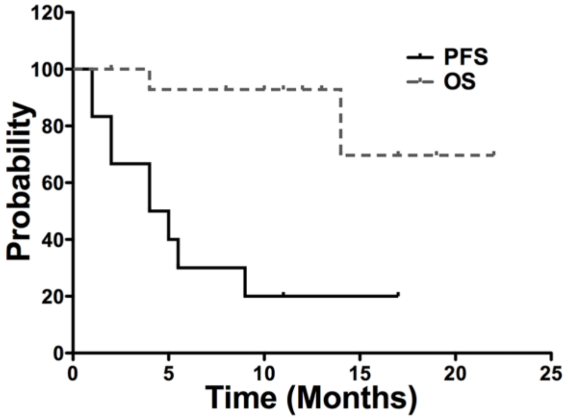

Figure S1: Kaplan-Meier curves of progression-free survival and overall survival.

The median progression-free survival is 10 months with a 95% CI of 5.9 to 12.9 months.

The median overall survival is 11 months with a 95% CI of 8.9 to 14.3 months.

Supplementary table

Table S1: Primer sequence for qRT-PCR and MSP.

Genes	Primer	Sequence (5' to 3')
GSTP1	Forward	AGGTCCTAGCCCCTGGCTGC
	Reverse	TCAGGGGAGGCCAGGAAGGC
CDKN2A	Forward	CTGGACACGCTGGTGGTGCT
	Reverse	CTATGCGGGCATGGTTACTGC
AKAP12	Forward	GGAGCCCTAAACAGCCAGGA
	Reverse	CTCCTCCTGCCCATCATCTG
MAGEA1	Forward	TCCGCCTTTCCCACTACCAT
	Reverse	TCCAGCATTTCTGCCTTTGT
MAGEA3	Forward	GGAGTCCGAGTTCCAAGCAG
	Reverse	AGGCAGGTGGCAAAGATGTA
GSTP1	M-Forward	TTCGGGGTGTAGCGCTCGTC
	M-Reverse	GCCCCAATACTAAATCACGACG
	U-Forward	GATGTTTGGGGTGTAGTGGTTGTT
	U-Reverse	CCACCCCAATACTAAATCACAACA
AKAP12	M-Forward	GTTGGCGCGTCGTAGCGTTT
	M-Reverse	TCCCCCCGATTTTTCCCTCG
	U-Forward	GAAATGGGAGGTGTTTGAGG
	U-Reverse	AACCCCAAACACACCCTACA
MAGEA1	M-Forward	AGGAGGGGATAAATATTTGGTTATAC
	M-Reverse	GCTCAAATCAATAAAAAAAACGTC
	U-Forward	AGGGGATAAATATTTGGTTATATGT
	U-Reverse	CACTCAAATCAATAAAAAAAACATC

M-Forward, M-Reverse: primers designed specifically for methylated DNA; U-Forward, U-Reverse: primers designed specifically for unmethylated DNA.
